# Clinical Characteristics and Prognostic Relevance of Different Types of Caregivers for Elderly Patients with Acute Heart Failure—Analysis from the RICA Registry

**DOI:** 10.3390/jcm11123516

**Published:** 2022-06-18

**Authors:** Manuel Méndez-Bailon, Noel Lorenzo-Villalba, Jorge Rubio-Garcia, María Carmen Moreno-García, Guillermo Ropero-Luis, Eduardo Martínez-Litago, Raúl Quirós-López, Sara Carrascosa-García, Alvaro González-Franco, Emmanuel Andrès, Jesús Casado-Cerrada, Manuel Montero-Pérez-Barquero

**Affiliations:** 1 Internal Medicine Department, Hospital Clínico San Carlos, Instituto de Investigación Sanitaria (IdISSC), Universidad Complutense, 28040 Madrid, Spain; manuelmenba@hotmail.com; 2 Service de Médecine Interne, Diabète et Maladies Métaboliques, Hôpitaux Universitaires de Strasbourg, 67000 Strasbourg, France; emmanuel.andres@chru-strasbourg.fr; 3 Internal Medicine Department, Hospital Clínico Universitario Lozano Blesa, 50009 Zaragoza, Spain; jorgerubiogracia@gmail.com; 4 Internal Medicine Department, Hospital de Manises, 46940 Valencia, Spain; mcmorenogarcia@gmail.com; 5 Internal Medicine Department, Hospital de la Serranía de Ronda, 29400 Malaga, Spain; guillermo.ropero.sspa@juntadeandalucia.es; 6 Internal Medicine Department, Hospital Santa Bárbara, 13500 Ciudad Real, Spain; emartinezl@sescam.jccm.es; 7 Internal Medicine Department, Hospital Costa del Sol, 29603 Málaga, Spain; quiroslopez77@gmail.com; 8 Internal Medicine Department, Consorcio Hospital General Universitario de Valencia, 46014 Valencia, Spain; saracarrascosagarcia@gmail.com; 9 Internal Medicine Department, Hospital Universitario Central de Asturias, 33011 Oviedo, Spain; alvarogfranco@yahoo.com; 10 Internal Medicine Department, Hospital Universitario de Getafe, 28905 Madrid, Spain; casadocerrada@telefonica.net; 11 Internal Medicine Department, IMIBIC, Hospital Universitario Reina Sofía, 14004 Córdoba, Spain; montero.manolo@gmail.com

**Keywords:** heart failure, caregivers, mortality, hospital readmission

## Abstract

Background: Patients with heart failure encompass a heterogeneous group, but they are mostly elderly patients with a large burden of comorbid conditions. Objective: The aim of this study was to compare the clinical characteristics and the prognostic impact on hospital admissions and mortality in a population of patients with HF with different types of caregivers (family members, professionals, and the patient himself). Methods: We conducted an observational study from a prospective registry. Patients from the National Registry of Heart Failure (RICA), which belongs to the Working Group on Heart Failure and Atrial Fibrillation of the Spanish Society of Internal Medicine (SEMI), were included. Patients with heart failure were classified, according to the type of main caregiver, into four groups: the patient himself/herself, a partner, children, or a professional caregiver. A bivariable analysis was performed between the clinical, analytical, therapeutic, and prognostic characteristics of the different groups. The endpoints of the study were all-cause mortality at 1 year; mortality at 120 days; and the readmission rate for HF at 30 days, 120 days, and 1 year of follow-up. In all cases, the level of statistical significance was set at *p* < 0.05. Results: A total of 2147 patients were enrolled in this study; women represented 52.4%, and the mean age was 81 years. The partner was the caregiver for 703 patients, children were caregivers for 1097 patients, 199 patients had a professional caregiver, and only 148 patients were their own caregivers. Women were more frequently cared for by their children (65.8%) or a professional caregiver (61.8%); men were more frequently cared for by their spouses (68.7%) and more frequently served as their own caregivers (59.5%) (*p* < 0.001). No statistically significant differences were observed in relation to readmissions or mortality at one year of follow-up between the different groups. A lower probability of readmission and death was observed for patients who received care from a partner or children/relative, with log-rank scores of 11.2 with *p*= 0.010 and 10.8 with *p* = 0.013. Conclusions: Our study showed that the presence of a family caregiver for elderly patients with heart failure was associated with a lower readmission rate and a lower mortality rate at 120 days of follow-up. Our study also demonstrated that elderly patients with good cognitive and functional status can be their own caregivers, as they obtained good health outcomes in terms of readmission and mortality. More prospective studies and clinical trials are needed to evaluate the impact of different types of caregivers on the outcomes of patients with heart failure.

## 1. Introduction

Heart failure (HF) is a chronic disease that is increasing worldwide. Patients with heart failure encompass a heterogeneous group, but they are mostly elderly patients with a large burden of comorbid conditions [1].

To reduce hospitalizations and mortality rates, it is recommended that patients with HF practice self-care, which also includes adherence to treatment. Self-care for HF is defined as the naturalistic decision-making process used by patients to maintain the stability of their disease (self-care maintenance), monitor signs and symptoms of HF (symptom awareness), and manage HF exacerbation (self-care). Evidence shows that HF self-care improves patient outcomes, such as the use of health care services and mortality. A recently published article demonstrated that worse self-care is an independent predictor of long-term mortality (both all-cause and cardiovascular), HF hospitalization, and the combination of these endpoints in patients with chronic HF [2]. Despite its positive effects, patients with HF have difficulty performing self-care.

In the self-care, management, and treatment of HF, the role of the caregiver is key, especially in patients with a profile of greater vulnerability due to their cognitive, functional, and social status, among other aspects. In this setting, most patients depend on support from relatives, friends, or some other external help in order to comply with medication and self-care. Thus, caregivers represent an important tool in the management of this group of patients. Both patients and caregivers must engage in medication management, adherence to diet and physical activity regimens, and symptom recognition [3,4,5]. Community nurses and other health care professionals also play important roles in HF care by optimizing the management, assessment, and evaluation of the patient’s clinical condition and care during transitions from the hospital to the home [6].

Some studies have demonstrated the effects of education of family caregivers at discharge on reducing hospital readmission in these patients [7]. However, the prognostic impact of different types of caregivers on the care of HF patients has not been evaluated [7]. The aim of this study was to compare the clinical characteristics and the prognostic impact on hospital admissions and mortality in a population of patients with HF with different types of caregivers (family members, professionals, and the patient himself/herself).

## 2. Methods

### 2.1. Design—Type of Study

We conducted an observational study from a prospective registry. Patients from the National Registry of Heart Failure (RICA), which belongs to the Working Group on Heart Failure and Atrial Fibrillation of the Spanish Society of Internal Medicine (SEMI), were included. The latter is a prospective, multicenter registry that has been active since 2008. It includes consecutive individual patients over 50 years of age with a diagnosis of HF at hospital discharge (acute decompensated or new-onset HF), according to European cardiology guidelines published in 2008.

### 2.2. Inclusion and Exclusion Criteria

Inclusion criteria: Subjects were included in the registry after hospital discharge and followed for at least one year. A total of 2147 patients were included. In the present analysis, we included patients older than 65 years who were registered from March 2008 to December 2020. Exclusion criteria: Patients who did not sign the informed consent to participate in the study were excluded.

### 2.3. Variables

We used personal history, physical examination, and clinical analysis records. Left ventricular ejection fraction (LVEF) as assessed by 2D echocardiography was included. The Charlson comorbidity index and Pfeiffer test were also collected. The Charlson comorbidity index predicts the one-year mortality for a patient who may have a range of comorbid conditions, such as heart disease, AIDS, or cancer (a total of 22 conditions are included). Each condition is assigned a score of 1, 2, 3, or 6, depending on the risk of dying associated with each one. Scores are summed to provide a total score to predict mortality. The Pfeiffer test is a short, reliable instrument used to detect the presence of intellectual impairment and determine its degree, if any.

Patients were classified, according to the type of main caregiver, into four groups: the patient himself/herself, partner, children, or a paid professional caregiver.

### 2.4. Statistical Analysis

Quantitative variables are expressed as means (standard deviation) and qualitative variables are expressed as absolute values (percentages). Quantitative variables were compared using ANOVA, and qualitative variables were compared using the Chi-square test. The post hoc Tukey method was used. Kaplan–Meier curves were constructed, comparing the groups using the log-rank test. A bivariable analysis was performed between the clinical, analytical, therapeutic, and prognostic characteristics of the different groups. The endpoints of the study were all-cause mortality at 1 year; mortality at 120 days; and the readmission rate for HF at 30 days, 120 days, and 1 year of follow-up. We performed a survival analysis for patients with HF at 120 days of follow-up with Kaplan–Meier curves. In all cases, the level of statistical significance was set at *p* < 0.05. Statistical analysis was performed using the IBM Statistical Package for Social Sciences (version 22.0, SPSS Inc., Chicago, IL, USA).

### 2.5. Ethical Aspects

The registry protocol was initially approved by the Ethics Committee of the Hospital Universitario Reina Sofía de Córdoba and was subsequently approved by each of the committees of the participating hospitals, code 18/349-E, with the last update approved by the CEIC on 9 August 2018. All patients signed an informed consent form prior to inclusion in the registry. The data were collected from a web page (www.registrorica.org, accessed on 1 March 2008) containing the anonymous database and accessed by each investigator through a personalized password. The registry’s design was previously published [8].

## 3. Results

A total of 2147 patients were enrolled in this study. Women represented 52.4% of patients, and the mean age was 81 years. The partner was the caregiver for 703 patients, children were the caregivers for 1097 patients, 199 patients had professional caregivers, and only 148 patients were their own caregivers. Hypertension and atrial fibrillation were seen in 88% and 54% of patients, respectively. The mean Barthel index was 81.2, and the mean Charlson score was 3.05. The mean left ventricular ejection fraction was 51.8% and was more frequently reduced for patients without a caregiver (44.7) (*p* < 0.001). Women were more frequently cared for by their children (65.8%) or a professional caregiver (61.8%); men were more frequently cared for by their wives (68.7) and more frequently served as their own caregivers (59.5%) (*p* < 0.001). (Table 1) In the latter case, the patients had a better functional status (Barthel index of 95) and cognitive situation (Pfeiffer of 0.5) than patients with other types of caregivers (*p* < 0.001). In relation to self-care, 1814 and 1555 patients followed low-sodium intake and weight monitoring regimens, respectively. Statistically significant differences were seen in relation to water restriction, which was lower for patients without an external caregiver (Table 1). The majority of patients were on beta blockers and ACE/ARA-2 inhibitors or anti-aldosterone agents. Statistically significant differences were observed in relation to the prescription of beta blockers (85.1%) and sacubitril valsartan (25.4%) in the group of patients without external caregivers (*p* < 0.01).

In relation to the endpoints analyzed, no statistically significant differences were observed in terms of readmission and mortality at 1 year of follow-up between the different types of caregivers. We did observe statistically significant differences in terms of readmission and mortality at 120 days, with lower rates of these events in patients with HF who had family members (child or partner) as their main caregivers (Table 1).

**Figure 1:**
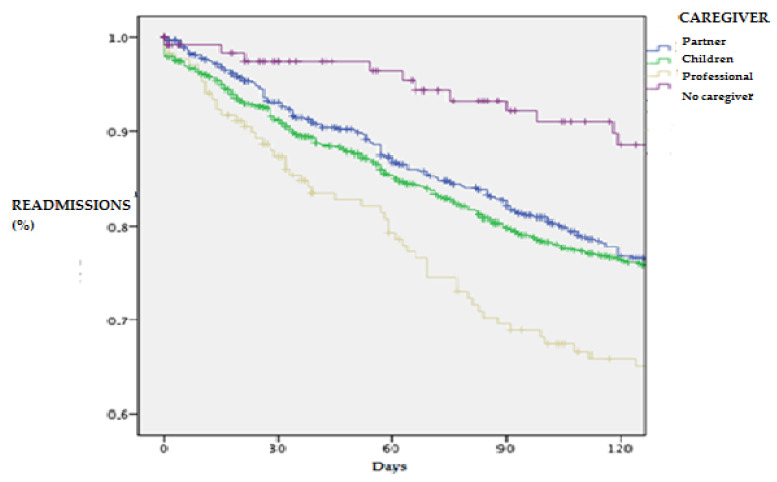
Survival analysis for readmissions in patients with HF according to caregiver type at 120-day follow-up.

**Figure 2:**
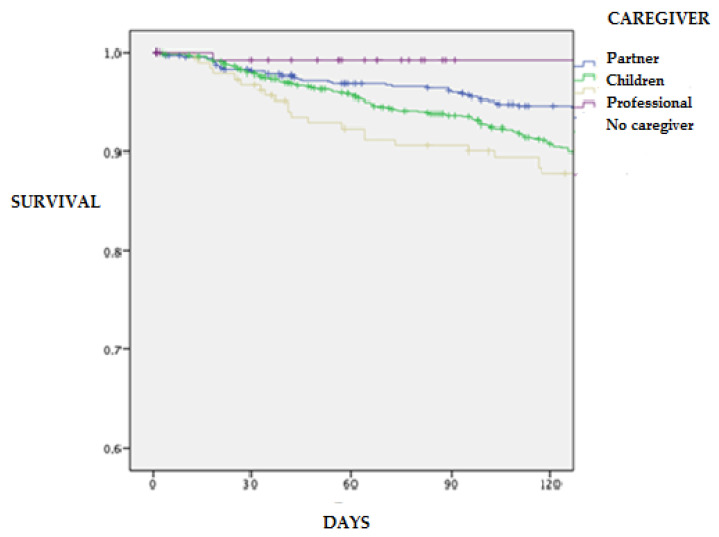
Survival analysis for mortality in patients with HF according to caregiver type at 120-day follow-up.

Figure 1 and Figure 2 show the tendency to present fewer admission and death events in these types of caregivers, with log-rank scores of 11.2 with *p* = 0.010 and 10.8 with *p* = 0.013.4

In Table S1 and Figure S1, we include the bivariate and Kaplan–Meier analysis for patients with heart failure, only considering the presence or absence of caregivers.

## 4. Discussion

The results of our investigation highlight that in our sample, HF patients who were admitted to the hospital and had a family caregiver had a more favorable prognosis regarding readmission and survival at 120 days of follow-up after hospitalization than patients with heart failure who were their own main caregiver or had a professional caregiver. This study is the largest of its kind in our country to evaluate the prognostic impact of the different types of caregivers on patients with HF admitted to hospitals. Our study demonstrates that elderly patients with good cognitive and functional status can also be their own caregivers, as they obtained good health outcomes in terms of readmission and mortality.

In relation to the characteristics of our series, we should highlight the important role played by family caregivers, initially the partners and later the children. This may be due to the characteristics of our aging population and the cultural aspects of our Spanish society; family values in the care of the elderly are deeply rooted in our country [9]. A low proportion of patients do not have caregivers, and this may be due to the fact that the great majority of elderly patients admitted to internal medicine services have a low capacity for self-care due to their high levels of dependency and cognitive deterioration [10]. In this sense, we emphasize that patients with HF who were their own main caregivers were younger and presented better cognitive and functional situations than the rest. Patients with HF for whom the caregiver was the patient had the smallest sample size, and it may be difficult to obtain solid conclusions in this regard. In relation to self-care, the patients in this group presented poorer adherence to measures such as control of water intake [11].

In relation to family caregivers, more than 60% of men received care from their wives, and more than 60% of women received care from their children or professional caregivers [12]. This may be due to the longer life expectancy of women in our country [13], which means that the main caregiver is less frequently the husband. The presence of a family caregiver in our study was accompanied by a favorable trend in terms of readmission and mortality in the short- and medium-term and attenuated at one year of follow-up. These findings may be due to the fact that the effects of family self-care have a higher impact in short- and medium-term follow-up than in long-term follow-up, in which the progressive evolution of the disease and the clinical situation of comorbidity and functional deterioration of the patient may lead to a higher risk of readmission and death [10]. In another study, the education levels of HF patients and caregivers were not correlated with readmission or mortality rates [14]. In the self-care measures evaluated, we only observed differences in relation to water restriction, which were in favor of patients with HF with caregivers, but we did not evaluate other measures recommended by clinical practice guidelines, such as self-adjustment of diuretics and monitoring of alarm signs [15].

The degree of clinical follow-up in heart failure programs carried out by each of the groups evaluated is also unknown. This study is limited by its retrospective nature and the fact that the RICA registry was not designed to evaluate the hypothesis of our investigation; the variables of self-care and the main caregiver were collected by the medical researchers, but no structured analysis of the patient’s self-care capacity—such as the European self-care scale—was carried out, and no evaluation of the degree of caregiver overload—such as the Zarit scale—was conducted [16]. In this research, variables related to the educational intervention received by the patient and caregivers were not collected either, which could affect the interpretation of the observed results. In this sense, there is a need of to perform specific prospective and randomized clinical trials to evaluate the impact of care and educational interventions by the patient himself/herself as well as by relatives and professional caregivers. The results of the MOTIVATE-HF trial have recently been published, showing that structured motivational interviewing with HF patients and caregivers may have an impact on patient survival. These findings could not be analyzed in our study considering its retrospective design, as motivational interviewing was not included as a variable in the RICA registry [17].

## 5. Conclusions

Our study showed that the presence of a family caregiver in elderly patients with HF was associated with a lower readmission rate and a lower mortality rate at 120 days of follow-up. More prospective studies and clinical trials are needed to evaluate the impact of different types of caregivers on the outcome of patients with HF.

## Figures and Tables

**Table 1:** Baseline characteristics and outcomes of patients with HF according to caregiver.

Variable	All (*n*= 2147)	Partner Caregiver (*n* = 703)	Children Caregiver (*n* = 1097)	Professional Caregiver (*n* = 199)	No Caregiver (*n* = 148)	*p*-Value
Age, median (SD)	81.06 (8.7)	77.28 (9.05)	83.63 (7.02)	83.53 (8.2)	76.6 * (10.7)	<0.001
Sex: male, *n* (%)	1022 (47.6)	483 (68.7) *	375 (34.2)	76 (38.2)	88 (59.5)	<0.001
Sex: female, *n* (%)	1125 (52.4)	220 (31.3)	722 (65.8) *	123 (61.8)	60 (40.5)	<0.001
Comorbidities						
Hypertension, *n* (%)	1889 (88)	604 (85.9)	989 (90.2)	176 (88.4)	120 (81.1) *	<0.001
T2DM, *n* (%)	993 (46.3)	367 (52.2)	467 (42.6)	82 (41.2) *	77 (52)	<0.001
COPD, *n* (%)	448 (20.9)	182 (25.9) *	203 (18.5)	34 (17.1)	29 (19.6)	0.001
Atrial fibrillation, *n* (%)	1172 (54.6)	361 (51.4)	629 (57.3)	119 (59.8)	63 (42.6) *	<0.001
Ischemic heart disease, *n* (%)	481 (22.4)	190 (27)	213 (19)	167 (16)	102 (31) *	<0.001
Pfeiffer index, median (SD)	1.5 (1.9)	1.08 (1.6)	1.31 (1.7)	2.02 (2.3)	0.5 * (1.09)	<0.001
Barthel index, median (SD)	81.2 (24.09)	89.2 (18.02)	75.3 (25.9)	73.8 (26.6)	95.9 * (9.7)	<0.001
Charlson score, median (SD)	3.05 (2.5)	3.2 (2.6)	3.02 (2.4)	2.9 (2.4)	2.6 * (3.2)	0.035
LVEF, median (SD)	51.8 (15.7)	50.3 (15.4)	53.3 (15.7)	54.5 (15.3)	44.7 * (15.3)	<0.001
Laboratory, *n* (%)						
Hemoglobin, (g/dL) median (SD)	12.09 (2.04)	12.3 (2.09)	11.9 (1.9)	11.8 * (1.9)	12.5 (2.2)	<0.001
Creatinine (ml/min/m^3^), median (SD)	1.3 (2.6)	1.2 (0.5)	1.4 (3.6)	1.2 (0.7)	1.2 (0.5)	0.692
proBNP (pg/mL), median	6654.6	5697.2	7108.06	7296.9	7555.1	0.058
Non-pharmacological treatment	19(0.88)					
Fluid restriction, *n* (%)	1365 (70.5)	417 (66.3)	741 (73.7)	124 (68.5)	83 (65.9)	0.008
Weight monitoring, *n* (%)	1555 (80)	497 (79.1)	803 (41.3)	153 (84.1)	102 (81)	0.510
Low-sodium diet, *n* (%)	1814 (93)	583 (92.4)	949 (93.9)	172 (94.5)	110 (88)	0.072
Pharmacological treatment, *n* (%)						
Beta blockers, *n* (%)	1522 (70.9)	516 (73.4)	753 (68.6)	127 (63.8)	126 (85.1) *	<0.001
ACE inhibitors/ARA-2, *n* (%)	1266 (59)	404 (57.5)	654 (59.6%)	131 (65.8) *	77 (52)	0.054
Sacubitril valsartan, *n* (%)	138 (6.4)	42 (6)	55 (5)	6 (3)	35 (25.4) *	<0.001
Anti-aldosterone agents	486 (22.6)	180 (37) *	233 (21.2)	38 (19.1)	35 (23.6)	0.099
Endpoints *n* (%)						
Mortality at 30 days, *n* (%)	546 (27.9)	155 (29.2)	299 (35.3)	67 (35.3)	25 (22.9) *	0.011
30-day readmission, *n* (%)	383 (19.7)	109 (17.3) *	201 (19.7)	49 (26.1)	24 (22.4)	0.053
Mortality at 120 days, *n* (%)	630 (32.1)	177 (27.8) *	341 (33.3)	76 (40)	336 (33)	0.010
120-day readmission, *n* (%)	691 (35.5)	207 (32.8) *	355 (34.8)	87 (46.3)	42 (39.3)	0.006
One-year readmission, *n* (%)	1365 (70.1)	430 (68.1)	718 (70.4)	142 (75.5)	75 (70.1)	0.279
One-year mortality, *n* (%)	1208 (61.6)	380 (59.7)	635 (62)	121 (63.7)	72 (66.1)	0.524

Legend: * adjusted residuals are outside the ranges +2 −2. T2DM: type 2 diabetes mellitus; COPD: chronic obstructive pulmonary disease; LVEF: left ventricular ejection fraction; ACE inhibitors: angiotensin-converting enzyme inhibitors; ARA-2: angiotensin II receptor antagonists.

## Data Availability

Data is contained within the article.

## Supplementary Materials

The following supporting information can be downloaded at: https://www.mdpi.com/article/10.3390/jcm11123516/s1. Table S1: Baseline characteristics and outcomes of patients with HF with and without caregivers; Figure S1: Analysis of survival at one year in patients with heart failure with and without caregivers.
