# Supplementary material for: Clinical Characteristics and Prognostic Relevance of Different Types of Caregivers for Elderly Patients with Acute Heart Failure—Analysis from the RICA Registry
Source: J Clin Med. 2022 Jun 18;11(12):3516. doi: 10.3390/jcm11123516 (PMC9225050; doi:10.3390/jcm11123516)

**Supplementary Material**

**Table S1**. Baseline characteristics and outcomes of patients with HF with and without caregiver.

| Variable | With Careviger (N=1999) | No Caregiver (N=148) | P value |
|---|---|---|---|
| Age media (sd) | 81.3 (8.4) | 76.6* (10.7) | <0.001 |
| Sex. male, N (%) | 934 (46.7) | 88 (59.5) | <0.002 |
| Sex female, N (%) | 1065 (53.3) | 60 (40.5) | <0.002 |
| **Comorbidities** | | | |
| Hypertension N (%) | 1769 (93.6) | 120 (81.1)* | 0.085 |
| T2DM N (%) | 916 (45.8) | 77 (52) | <0.001 |
| COPD N (%) | 419 (21) | 29 (19.6) | 0.392 |
| Atrial fibrillation N (%) | 1109 (55.5) | 63 (42.6)* | <0.002 |
| Ischemic Heart disease N (%) | 435 (21.8) | 102 (31)* | <0.007 |
| Pfeiffer index median (SD) | 1.6 (1.9) | 0.5* (1.09) | <0.001 |
| Barthel index median (SD) | 80.1 (24.4) | 95.9* (9.7) | <0.001 |
| Charlson score median (SD) | 3.08 (2.5) | 2.6* (3.2) | 0.031 |
| LVEF median (SD) | 52.4 (15.6) | 44.7* (15.3) | <0.001 |
| Laboratory N (%) | | | |
| Hemoglobin (g/dl) median (SD) | 12.05 (2.02) | 12.5 (2.2) | <0.001 |
| Creatinine (ml/min/m$^3$) median (SD) | 1.3 (2.7) | 1.2 (0.5) | 0.692 |
| proBNP pg/ml median | 6598.6 | 7555.1 | 0.058 |
| Non pharmacological treatment | | | |
| Fluid restriction N (%) | 1282 (70.6) | 83 (65.9) | 0.154 |
| Weight monitoring N (%) | 1453 (80) | 102 (81) | 0.443 |
| Low sodium diet N (%) | 1704 (93.4) | 110 (88) | 0.022 |
| Pharmacological treatment N (%) | | | |
| Beta blockers N (%) | 1396 (70.9) | 126 (85.1) | <0.001 |
| ACE inhibitors/ARA-2 N (%) | 1189 (59) | 77 (52) | 0.046 |
| Sacubitril valsartan N (%) | 103 (5.2) | 35 (25.4) | <0.001 |
| Anti-aldosterone agents | 451 (22.6) | 35 (23.6) | 0.414 |
| **Endpoints N (%)** | | | |
| Mortality at 30 days | 521 (28.1) | 25 (22.9) | 0.142 |

| | | | |
|---|---|---|---|
| N (%) | | | |
| 30 days readmission<br>N (%) | 359 (19.5) | 24 (22.4) | 0.266 |
| Mortality at 180 days<br>**N (%)** | 594 (32.1) | 36 (33) | 0.010 |
| 180 days readmission<br>N (%) | 649 (35.3) | 42 (39.3) | 0.232 |
| One year readmission<br>N (%) | 1290 (70.1) | 75 (70.1) | 0.533 |
| One year mortality<br>N (%) | 1136 (61.6) | 72 (66.1) | 0.191 |

**Figure S1**. Analysis of survival at one year in patients with heart failure with and without caregivers. Log Rank (Mantel-Cox) 10,830 1 p<0.001.

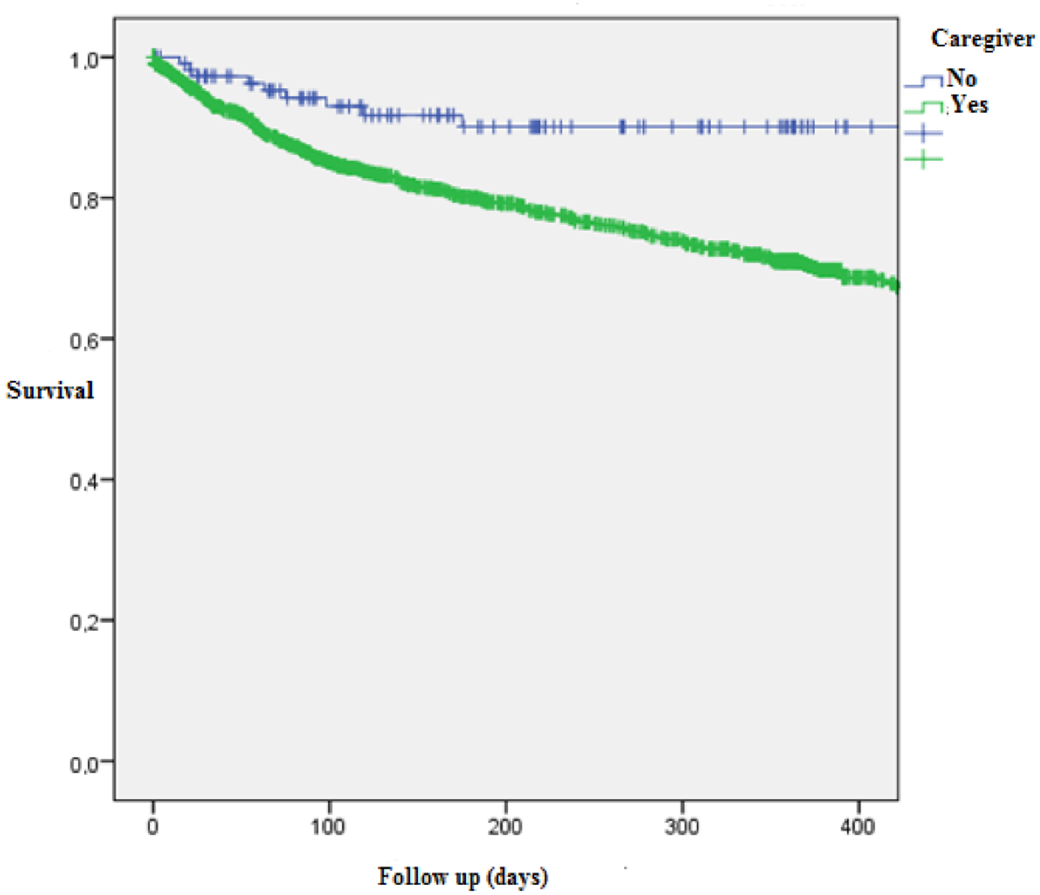

Supplement: Supplementary file 1 [file jcm-11-03516-s001.zip › jcm-1698798-supplementary.pdf]
